# Improvement of White Spruce Wood Dimensional Stability by Organosilanes Sol-Gel Impregnation and Heat Treatment

**DOI:** 10.3390/ma13040973

**Published:** 2020-02-21

**Authors:** Diane Schorr, Pierre Blanchet

**Affiliations:** 1 FPInnovations, 1055 rue du Peps, Quebec City, QC G1V 4C7, Canada; 2 NSERC Industrial Research Chair on Ecoresponsible Wood Construction (CIRCERB), Forest and Wood Sciences Department, Université Laval, 2425 rue de la Terrasse, Quebec City, QC G1V 0A6, Canada; pierre.blanchet@sbf.ulaval.ca

**Keywords:** dimensional stability, spruce wood, organosilanes, cell wall

## Abstract

Wood is a living material with a dimensional stability problem. White spruce wood is a Canadian non-permeable wood that is used for siding applications. To improve this property, white spruce wood was treated with organosilanes sol-gel treatment with different moisture content (oven dried, air dried, and green wood). No major morphological changes were observed after treatment. However, organosilanes were impregnated into the cell wall without densifying the wood and without modifying the wood structure. Si-O-C chemical bonds between organosilanes and wood and Si-O-Si bonds were confirmed by FTIR and NMR, showing the condensation of organosilanes. The green wood (41% moisture content) showed only 26% dimensional stability due to the presence of too much water for organosilanes treatment. With a moisture content of 14%–18% (oven dried or air dried wood), the treatment was adapted to obtain the best improvement in dimensional stability of 35% and a 25% reduction of water vapor sorption. Finally, impregnation with organosilanes combined with the appropriate heat treatment improved the dimensional stability of white spruce wood by up to 35%. This treated Canadian wood could be an interesting option to validate for siding application in Canada.

## 1. Introduction

The demand for wood material is decreasing for sidings in America, therefore, it is necessary to find a solution to revitalize this material [1]. In Quebec (Canada), white spruce wood (*Picea glauca* (M). V.), is an important species used in sidings; however, one of its most known problems is its dimensional stability. Because of its hygroscopic behavior, wood is prone to crackling, warping, and fading due to dimensional variation, which is a problem in siding applications. Wood modification is necessary to improve these physical properties and to guarantee this material for siding applications in Quebec. 

There are two main wood modifications used to improve its dimensional stability.

Heat and oleothermal treatments are the first main wood modifications. These heat treatments allow destruction and reorganization of the wood structure by reducing the hydroxyl content [2]. These treatments are widely used in Europe; however, this process is quite variable depending on the species. Few studies have shown improvement in the dimensional stability of white spruce wood (*Picea glauca (M). V.*) by torrefaction (heat process without oxygen). However, it has shown some good results, with an improvement of up to 43% in the dimensional stability of spruce wood [3,4,5]. In the literature, densification has even been coupled with torrefaction treatment to improve the dimensional stability of spruce wood [6,7]. However, these high-temperature treatments reduced the mechanical properties and the coating adhesion required for the wood to be used as a siding. Currently, torrefied wood is not preferred in Quebec (Canada) for wood siding applications.

Chemical treatment is the second main wood modification. There are two main processes.

- One is to fill the empty spaces in the wood, this is densification using thermosetting resins that could react under the temperature in the wood structure and would block the accessibility of the hydroxyl groups. Some densified woods with better dimensional stability are commercialized in Europe (Kebony© and Belmadur©) by using permeable species such as raiata pine (*Pinus Radiata* D. Don). In the literature, densification has also been combined with nanomaterials to improve the physical properties of sugar maple (*Acer saccharum*, S.M) and red oak (*Quercus Rubra*, R.O) [8]. However, these densifications could not be applied to softwood Canadian species such as the white spruce used for siding applications due to its low permeability. In addition, densified wood is not used for siding application in Quebec because of the nailing system used.
- The other chemical treatment is the use of a chemical bond. In fact, chemical products could react to form some chemical bonds with the wood’s hydroxyls groups [9]. Some commercially modified woods are already known to have better dimensional stability, such as Accoya titan wood©. However, they are only used on permeable species such as radiata pine (*Pinus Radiata* D. Don). In the literature, some authors observed an improvement of up to 64% in the dimensional stability of spruce wood by acetylation using microwaves [10]. Pepin et al. worked on treating white spruce with organic fungicides, and dimensional stability increased by 27% [11].

The new emergence concerns nanomaterials. Indeed, several studies have worked on the addition of nanomaterials [12,13,14].

Another chemical treatment could be of interest to treat white spruce wood for siding applications and to improve dimensional stability. 

Organosilanes have already been used in plastic agents, textiles, and coatings. They can be used to make the material more hydrophobic [15,16,17]. Hydrolyzed and monomeric organosilanes were impregnated into wood pine (*Pinus sylvestris* L.) and birch (*Fagus sylvatica* L.) sapwood [18]. The authors observed that hydrolyzed silanes favored lumen filling while monomeric organosilanes were impregnated more into the cell walls with a lower mass gain. Dimensional stability was improved by up to 30% for all the organosilanes studied (Tetraethylorthosilicate (TEOS), triethoxymethysilane (PTEOS), and methyltryethoxysilanes (MTES)). Dimensional stability improvement, hydrophobicity, resistance to fungi, or even fire resistance were observed in treated wood when using organosilanes groups containing functional groups such as the aromatic cycle [19] and amine groups [20]. However, when adding these features, the chemical product is more expensive. In using methyltrimethoxysilane (MTMOS), interesting results were observed on Japanese cedar sapwood (*Cryptomeria jopanica* D. Don) [21] or with green elm (*Ulmus L.*) [22]. Indeed, treated wood showed a 75% decrease in water absorption and an improvement in dimensional stability of 72%–80%. Thermal properties have also been improved for Japanese cedar. Other authors have studied sol-gel treatment of wood by MTMOS with a titanium oxide solution. They also observed a decrease in water absorption of 25% for titanium oxide and 123% for MTMOS. For water vapor adsorption, it decreased by 72% for TiO2 and 138% for MTMOS [23].

In this study, the objective was to impregnate white spruce wood to improve its dimensional stability for use in siding applications. For this purpose, organosilanes have been used to chemically modify white spruce wood, which should improve its dimensional stability. Different moisture contents of white spruce wood and different post-treatments were used to find a process to obtain the best dimensional stability of the treated white spruce wood. The wood microstructure, chemistry, and physical properties (dimensional stability, water vapor sorption) of the treated wood were also studied.

## 2. Materials and Methods

### 2.1. Materials

The oven dried white spruce (OWS) wood, 14% moisture content, was obtained from Maibec in Saint-Romuald. The air dried white spruce wood (AWS), 19% moisture content, was obtained from Bionor in Rouyn-Noranda. Since green spruce wood (GWS) was not found, it was decided to soak the air-dried spruce wood in water at 20 °C with a wood moisture content of 41%. Wood samples were from knot-free sapwood. Ethanol (85%) was bought from Labmat in Quebec, Canada, acetic acid and methyltrimethoxysilane were obtained from Millipore Sigma.

### 2.2. Organosilanes Sol-Gel Preparation

Organosilanes solution was prepared using methyltrimethoxysilane, acetic acid, and ethanol at a molar ratio of 0.12/1/0.005 (solution A) and 0.24/1/0.005 (solution B). Methyltrimethoxysilane was first mixed with ethanol, and then acetic acid was added. The pH was adjusted to 3 with an acid solution of HCl. Finally, the solution was mixed at 60 °C for a period of 1 h 30 min.

### 2.3. Wood Treatment

The oven-dried wood samples were conditioned before impregnation at 20 °C and 65% relative humidity (RH). The air-dried wood and green wood samples were kept in a freezer at −21°C. Before impregnation, these samples were placed at 20 °C and 80% RH to be defrosted. Before impregnation, samples were cut to 20 × 20 × 19 mm and were weighted and measured in radial and tangential directions. The longitudinal direction was not noted as it did not change significantly after impregnation. White spruce samples were immersed in solution (A or B) in a vessel for impregnation. Vacuum was applied for 15 min at 25 mm Hg. A pressure of 620 kPa was applied in the vessel for a period of 1 h.

After impregnation, different post-treatment steps were performed (Table 1):

- The first step was daytime storage at 80% RH; the idea for this was to hydrolyse the organosilanes before the heat treatment.
- The second step was the heat treatment:
  ○ A heat treatment of 95 °C for 24 h to condense the organosilanes with themselves and the wood.
  ○ After the first result, the heat treatment test was modified by performing the first step at 50 °C for 24 h and then at 103 °C for 18 h. The purpose of this was to evaporate the solvent (ethanol) at 50 °C and then increase the temperature to 103 °C for the organosilanes condensation. An increase of temperature was expected to intensify the condensation of organosilanes within the wood (OWSSiT2 and OWSSiHT2).

**Figure 1:**
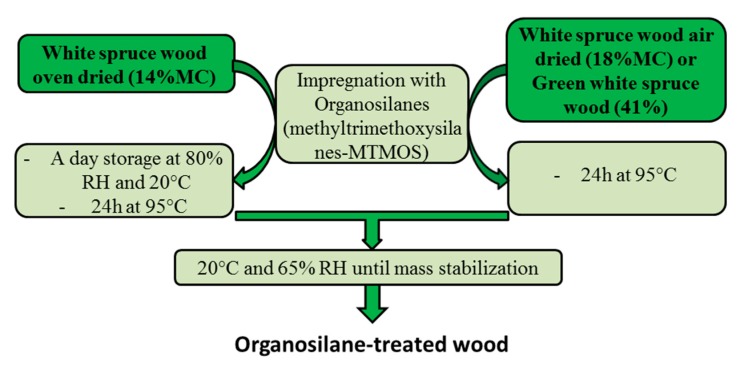
Flow diagram of the experimental procedure.

The flow diagram of the experimental procedure is depicted in Figure 1.

After the post-treatment, samples were placed at 20 °C and 65% RH until mass stabilization.

### 2.4. Wood Characterization

The wood chemical characterization was obtained from FTIR spectra and NMR ^13^C and ^29^Si spectra. FTIR spectra were recorded between 4000–400 cm^−1^, at 4 cm^−1^ of resolution using 64 scans. The baseline was obtained on all spectra, and normalization was done on the peak at 1515 cm^−1^ (band characteristic of lignin aromatic). The organosilane-treated wood spectrum was subtracted from the heat-treated wood spectrum. Solid-state cross polarization magic angle spinning (CP/MAS) ^13^C-NMR spectra were obtained at 75.3 MHz using Bruker Avance NMR equipment (Bruker Biospin Canada, Milton, ON). Wood samples were finely ground and placed in 7 mm zirconia rotors and spun at 5 kHz. The spectra were acquired at 75 MHz, a FID of 2048 dots, a recycled delay of 4 s, and a contact time of 1 ms for a total of 4096 scans. A TOSS B sequence was used to eliminate rotation bands. Solid-state CP/MAS ^29^Si-NMR spectra were obtained at 59 MHz using Bruker Avance NMR equipment (Bruker Biospin Canada, Milton, ON, Canada). Wood samples were also finely ground and placed in a 7 mm zirconia rotor and spun at 5 kHz. The spectra were acquired at 59 MHz, with a contact time of 20 ms, a FID of 1024 dots, and a recycling delay of 4 s for a total of 4096 scans. 

Morphology of non-treated and treated wood was observed by scanning electron microscopy (JEOL model JSM-840A, Peabody, MA, USA). A sample slice was cut with microtome. The surface was coated with gold and palladiums to facilitate the observation by SEM. Parameters were an accelerated voltage at 15 kV and a magnitude at ×100 or ×500. SEM was coupled with energy dispersion by X-ray (EDX) to locally analyze non-organic material such as silica.

### 2.5. Dimensional Stability 

To study wood dimensional stability, 15 samples per treatment were conditioned at 20 °C and 65% RH before the test. Their mass, radial, and tangential dimensions were measured. The dimensional stability test was obtained by combining the methods in two norms (ISO 4469, ISO 4859). First, samples were placed in the oven at 103 °C until the mass became stable. The samples were then placed in a desiccator and measured (radial, tangential dimension, and mass) after being at room temperature. Samples were placed for 2 days in 20 °C and 65% RH and then immerged in distilled water at 20 °C until the mass became constant. Samples were swiped and measured in radial and tangential dimensions. These two steps constituted one cycle. The study was conducted for three cycles to see whether the treatment remained effective for all cycles or whether any leaching decreased performance throughout the cycles. Here are the equations to calculate the swelling and shrinkage of the samples.

Swr (%) = [(R1 − R0)/R1]*100(1)

Swt (%) = [(T1 − T0)/T1]*100(2)

Shr (%) = [(R1 − R0)/R1]*100(3)

Sht (%) = [(T1 − T0)/T1]*100(4)

With Swr and Swt being the swelling coefficient in the radial and tangential directions, respectively and Shr and Sht being the shrinkage coefficient in the radial and tangential directions, respectively. R0 and T0 are the radial and tangential dimensions (mm), after the shrinkage test and R1 and T1 are the radial and tangential dimensions (mm), respectively, after the swelling test.

To calculate the dimensional stability, here are the equations used:

ASE Swr (%) = [(Swr0 − Swr1)/Swr0]*100(5)

ASE Swt (%) = [(Swt0 − Swt1)/Swt0]*100(6)

ASE Shr (%) = [(Shr0 − Shr1)/Shr0]*100(7)

ASE Sht (%) = [(Sht0 − Sht1)/Sht0]*100(8)

Swr, Swt, Shr, and Sht are, respectively, the swelling radial and tangential coefficient and the shrinkage radial and tangential coefficient.

ASE Swr, ASE Swt, ASE Shr, and ASE Sht are, respectively, the dimensional stability coefficient of radial and tangential swelling and the radial and tangential shrinkage.

### 2.6. Vapor Sorption Analysis 

Vapor sorption was studied using a water vapor sorption analyzer (VTI-SA) from TA Instruments, New Castle, DE, USA. Sample dimensions were 3 × 3 mm. A humidity ramp from 0% to 95% humidity with several steps (0%; 5%; 20%; 40%; 60%; 80%; 95%; 80%; 60%; 40%; 20%; 5%) was programmed to evaluate water adsorption and desorption. The mass equation for the maximal mass at 95% humidity is as follows:

EMC = 1 − (M2/M0)(9)

EMC = Equilibrium moisture content; M2 = sample mass at 95% RH and M0 = dried sample mass at 0% RH.

## 3. Results and Discussion

### 3.1. Effect of Organosilanes Treatment Depending on Wood Moisture Content

#### 3.1.1. Mass Gain, Dimensions Changes of Organosilanes-Treated Wood 

Oven dried white spruce wood (Moisture content (MC):14%) treated with organosilanes (OWSSiT and OWSSiHT), shows a mass gain of 11%–13% compared to raw wood and 15% compared to wood with heat treatment alone-OWST and OWSHT. For the sample size after treatment, treated wood with organosilanes showed slightly less shrinkage in both directions than wood treated only with heat treatment (0.3%–0.5%). For air-dried wood treated with organosilanes (AWSSiT and AWSSiHT) compared to wood with heat treatment alone (AWST and AWSHT), the weight gain was the same as for oven dried wood (15%). In terms of sample size, there was less shrinkage in the radial (0.5%) and especially tangential direction (1.5%) compared to heat-treated wood and even slightly less shrinkage (0.5%) compared to air-dried white spruce wood. Finally, for green wood, the final mass loss of wood treated with organosilanes (GWSSiT and GWSSiHT) is much lower than heat-treated wood (by 10% and 14%). In terms of dimensions, the shrinkage was slightly less important for the tangential (0.7%–1%) and especially for the radial direction (1%–1.5%). Finally, all treatments with organosilanes resulted in weight gain and a slight swelling in radial and tangential directions (regardless of moisture content) compared to wood treated only with the same heat treatments. The dimension changes could be explained by the organosilanes impregnated in the wood cell wall.

#### 3.1.2. Wood Morphology and Chemistry Study after Organosilanes Impregnation

**Figure 2:**
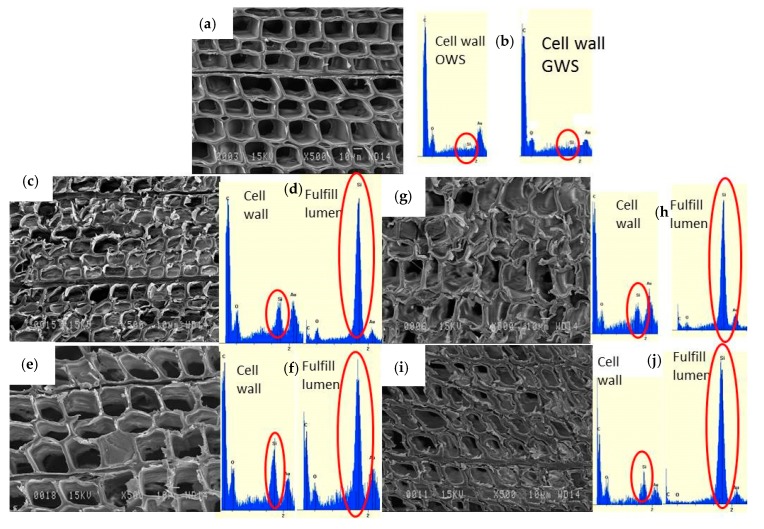
SEM images of (**a**) oven dried white spruce wood (OWS); (**c**) oven dried white spruce wood with SiT treatment; (**e**) oven dried white spruce wood with SiHT treatment; (**g**) green white spruce wood (GWS) with SiT treatment; (**i**) green white spruce wood with SiHT treatment; EDX spectra with silica peaks for (**b**) oven dried and green white spruce, (**d**) OWS with SiT treatment in the cell wall and lumen; (**f**) OWS with SiHT treatment in the cell wall and lumen; (**h**) GWS with SiT treatment in the cell wall and lumen; (**j**) GWS with SiHT treatment in the cell wall and lumen.

The wood microstructure was observed by scanning electron microscopy (Figure 2). Air-dried wood (18% humidity) and oven-dried wood (14% MC) show the same results and images. However, some differences are observed with green wood (41% MC). All the images show that the process using organosilanes is indeed a process promoting the penetration of organosilanes into the cell wall and not wood densification by filling the lumens with the organosilanes (Figure 2c–i) [18,24]. However, some lumens are filled with organosilanes, but only in a minority way. The wood microstructure remains intact, especially for the SiHT process, including the wet step at 80% humidity (Figure 2e,i). For green wood, the cells seem to be more deformed due to more intense drying (41% MC wood, initially), especially for the GWSSiT treatment (Figure 2g).

Using X-ray dispersive energy micro-analysis, raw white spruce wood shows no presence of silica in its cell wall (Figure 2b). In samples treated with organosilanes, silica is distributed homogeneously in the cell wall (Figure 2d,f,h,j) as described in other studies [16,17,18]. Few lumens are clogged due to the condensation of organosilanes in the lumen. The presence of organosilanes is confirmed by the results of the EDX showing a high response of silica in these filled lumens, greater than that of carbon or oxygen.

**Figure 3:**
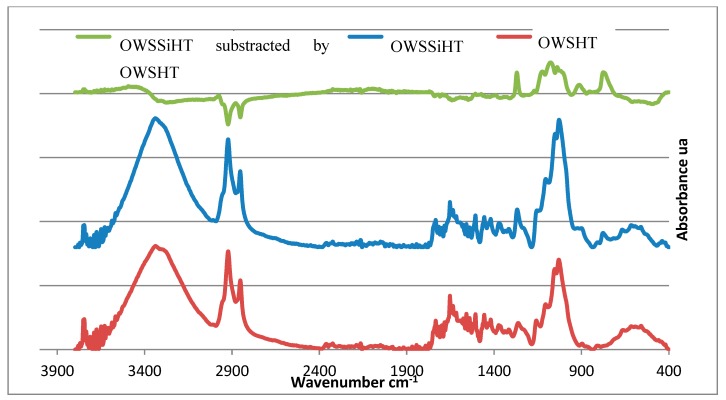
Oven dried treated wood (OWSSiHT), oven dried heated wood (OWSHT), and OWSSiHT subtracted by OWSHT FTIR Spectra.

Figure 3 shows the spectra of heat-treated wood (OWSHT) (red), Organosilanes-treated wood with the same heat treatment (OWSSiHT) (blue), and the subtraction spectrum after normalization between the spectrum OWSSiHT and OWSHT (green). The spectra (blue and red) show a similar spectrum with small differences in the wood structure with some additional peaks. Indeed, the spectrum of organosilanes-treated wood subtracted from the spectrum of heat-treated wood shows new peaks between 1400 and 800 cm^−1^. The peak at 1274 cm^−1^ is characteristic of the Si-CH_3_ bond; peaks at 1139 and 1060 cm^−1^ come from the stretching of the Si-O-Si bonds, and the peaks at 783 and 447 cm^−1^ come from the stretching of the bonds Si-O; Si-O-C; and Si-C [20,25]. The subtracted spectrum shows changes in hydroxyl functions. Indeed, above 3400 cm^−1^, there is the presence of hydroxyls from organosilanes. The presence of hydroxyls with organosilanes is also confirmed by the peak at 933 cm^−1^. Organosilanes may not have totally reacted. On the other hand, below 3400 cm^−1^, there is a decrease in the band corresponding to the hydroxyls stretching from the wood. A surprising fact is the decrease of some characteristic wood peaks. The decreases in alkane peaks (2900–2800 cm^−1^) and aromatic cycles (1700–1550 cm^−1^) can be explained by the solvent used. Indeed, ethanol could solubilize some wood extractives (polyphenols) [26], leading to a decrease of certain peaks on the infrared spectrum compared to heat-treated wood only.

**Figure 4:**
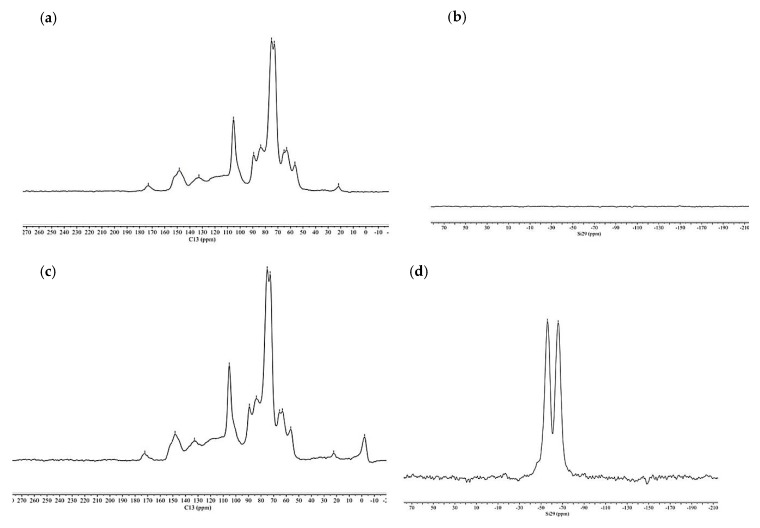
^13^C and ^29^Si NMR spectra of oven dried non-treated wood (**a**,**b**) and organosilanes-treated oven dried wood (**c**,**d**).

Figure 4 shows the ^13^C NMR and ^29^Si spectra of oven dried wood, non-treated and treated with organosilanes (OWSSiHT).

In the ^13^C NMR spectra, the characteristic peaks of cellulose and hemicelluloses are at 105.3 ppm for C1 carbon and at 89.2 ppm and 83.6 ppm for C4 of cellulose crystalline and amorphous, respectively. The C2, C3, and C5 of hemicelluloses are present in the peak at 75 ppm, and those of cellulose are present in the peak at 72.8 ppm. The C6 of crystalline cellulose and C5 of hemicelluloses have a peak at 65.0 ppm. The C6 amorphous of cellulose is presented in the peak at 63.0 ppm [27,28,29]. The peaks from lignin are present at 173.1 ppm for the carboxyl groups, 148.1 ppm for the C4 carbon atoms, 132.8 ppm for the aryl group, 89.2 ppm for the βO4 and αO4 bond of lignin, and 56.4 ppm for the carbon from the methoxyl groups of lignin. The peak at 21.7 ppm corresponds to the methyl groups of wood acetyl groups from cellulose and hemicelluloses. For the organosilanes-treated wood, the peaks are very similar; however, some chemical shifts have been changed due to the condensation created between the organosilane hydroxyls and cellulose, hemicelluloses, and lignin hydroxyls. Indeed, peaks at 173 ppm, 65 ppm, and 21.7 ppm shifted by 0.3–1 ppm due to the reaction between organosilanes and the wood. Moreover, a new peak at −2.2 ppm comes from the methyl groups of organosilanes present in the wood.

**Figure 5:**
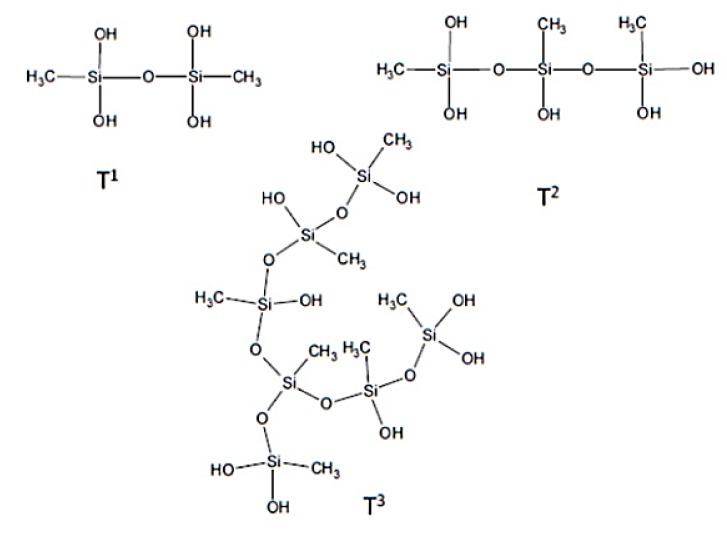
Different forms of organosilanes (from MTMOS) bonded to each other.

For ^29^Si spectra (Figure 4), the non-treated wood spectrum shows no peaks due to the absence of silica in the wood. MTMOS signals in the ^29^Si NMR spectrum are at –37 and –39 ppm [30]. The organosilane-treated wood spectrum does not show these peaks, which means that no MTMOS remained unreacted. However, this spectrum shows two different peaks. One at –56.0 ppm, which corresponds to the T2 form of the organosilanes (siloxane linear groups with two siloxanes bond), and the other peak at –65.9 ppm, which corresponds to the T3 form of the organosilanes (complex cross linked structure with the three siloxanes bonds). The T1, T2, and T3 forms of organosilanes are presented in Figure 5 [28,31].

#### 3.1.3. Dimensional Stability and Water Vapor Sorption of Organosilane-Treated Wood 

Dimensional stability tests were performed on untreated control wood, heat-treated wood, and organosilane-treated wood to study the effect of heat treatment and organosilanes treatment compared to raw wood. Three cycles of swelling and shrinkage were performed to observe whether the treatment was maintained over time (even after possible leaching). In terms of swelling or shrinkage, the same variations were observed. Untreated control wood with different moisture content had a shrinkage of 4.5% to 5.1% and 7.5% to 8.9%, respectively in the radial and tangential directions, creating a volumetric shrinkage of 12.8%–13.4%. This is consistent with the literature, which speaks of about 13% volumetric shrinkage [32,33]. For treated samples, radial shrinkage (or swelling) variations ranged from 2% for organosilane-treated wood to 4.8%–5% for heat-treated wood. Tangential shrinkage or swelling, which is the most important direction for dimensional variation, ranges from 4.9% for wood treated with organosilanes to 8% for heat-treated wood. These results generally show similarities as the cycle progresses. There is small variation between the different cycles for conventional kiln-dried and air-dried spruce (19%), showing that the treatment was still effective after several cycles. On the other hand, green white spruce wood shows more variation in the different cycles and differences between cycles. This could be the effect of inhomogeneity based on the high wood moisture content (41%) before treatment. 

**Figure 6:**
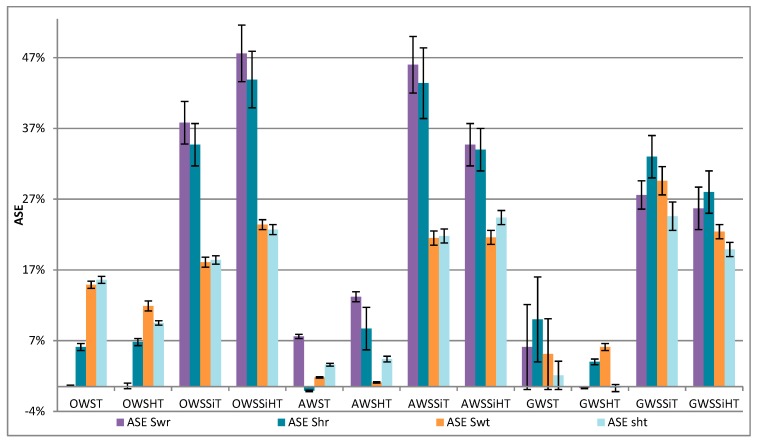
Anti swelling (ASE Sw r and ASE Swt) or shrinkage (ASE Sh r and ASE Sht) efficiency (dimensional stability) in the radial direction (r) and in the tangential direction (t) for treated OWS, AWS, and GWS using all the different treatments.

Figure 6 shows the dimensional stability (expressed as a percentage compared to untreated wood) for swelling and shrinkage in radial and tangential directions. 

Heat treatments alone (T and HT) for all RH (OWS, AWS, and GWS) show a slight improvement in dimensional stability of less than 15% in all directions for either swelling or shrinkage. Heat treatments for 14% oven-dried wood (OWST) have an effect on the tangential direction (SD Gt: 14% maximum), whereas with air-dried wood (AWST) or green wood (GWST), these heat treatments have more effect on the radial direction (SD Gr: 13% maximum) and a smaller effect in the tangential direction. For oven-dried white spruce wood, it was found that treatment with organosilanes with the wet step (OWSSiHT) gave better results (SD Gr and Rr: 45% and Gt and Rt: 22.5%) than without this step (OWSSiT) (35.8% in the radial direction and 17.7% in the tangential direction). The presence of water vapor hydrolyzes the organosilanes, then their condensation was facilitated with the wood hydroxyl during the heat treatment [34]. With regard to air-dried wood (AWS) or green wood (GWS), the wet step was not necessary as the results decreased with this step. This is due to the initial presence of water in the wetter and greener wood. Wood with higher moisture content allowed the organosilanes to hydrolyze in the wood without the wet treatment. This step is then unnecessary and even slightly reduces the results due to the possible leaching of organosilanes during this step. An interesting fact is that, depending on the initial wood (kiln-dried or air-dried), the dimensional stability results will be the same if the post-treatment is adapted for each of them. Indeed, organosilanes oven-dried wood will undergo a wet treatment before its heat treatment, whereas organosilanes air-dried or green wood will only have to be heat-treated without the prior wet step.

As a conclusion of this test, the best dimensional stability (radial and tangential) is 35% for oven-dried wood (OWSSiHT), 32% for air-dried wood (AWSSiT), and 27% for green wood (GWSSiT). These results are similar and even better than using the nano-SiO_2_ particles, which obtained a maximum dimensional stability of 27% for pine and beech [35] or 31% with MTMOS for Araucaria angustifolia [36]. 

These results (independently from initial wood humidity) showed that the treatments had a better effect on dimensional stability in the radial direction than in the tangential one. However, wood deformations occurred mainly in the tangential direction [37], so treatments should be improved to gain dimensional stability in the tangential direction.

Table 2 shows the maximum adsorption results of water vapor at 95% humidity. Heat treatments have no or very small effects on the adsorption of water vapor. 

As for dimensional stability, the best treatment for oven-dried wood treated with organosilanes is with the wet step (OWSSiHT). However, the difference is small. For air-dried or green spruce wood, there is little difference between the two heat treatments (OWSSiHT and OWSSiT). A maximum reduction in water vapor adsorption of 25% was achieved, compared to the control wood, for oven-dried spruce wood treated with OWSSiHT treatment.

### 3.2. Modification of Organosilanes Treatment on Oven Dried White Spruce Wood 

Treatment modifications were performed to improve dimensional stability, especially in the tangential direction. The tests were conducted only on oven-dried white spruce wood at 14% MC due to its better dimensional stability obtained with the organosilanes treatment.

- The concentration of organosilanes was doubled to ensure the reaction of a maximum of wood hydroxyls (OWSSi2T and OWSSi2HT)
- The heat treatment was modified by performing a first step at 50 °C for 24 h and then a second step at 103 °C for 18 h. The purpose being the evaporation of the solvent (ethanol) at 50 °C then at 103 °C for the condensation of the organosilanes. An increase of the temperature was supposed to intensify the condensation of the organosilanes with the wood (OWSSiT2 and OWSSiHT2)
- A combination of these two modifications (increase of the concentration and modification of the heat treatment after impregnation) (ESSi2T2 and ESSi2HT2) was used to treat the wood

To evaluate the treatment’s efficiency, a comparison was made with the first organosilanes treatments (OWSSiT and OWSSiHT); the treatment did not provide additional mass gain by doubling the organosilanes concentration. In terms of dimensions, there was no significant difference between treatments. With the modified heat treatment as well as the combination of these two treatments, a decrease in mass was observed, compared to the initial treatment with the organosilanes (OWSSiT ou OWSSiHT). This is due to a more intense and longer heat treatment. However, in terms of dimensions, the new heat treatments (OWSSiT2, OWSSiHT2, OWSSi2T2, and OWSSi2HT2) involved more wood swelling than the initial heat treatment (95 °C).

**Figure 7:**
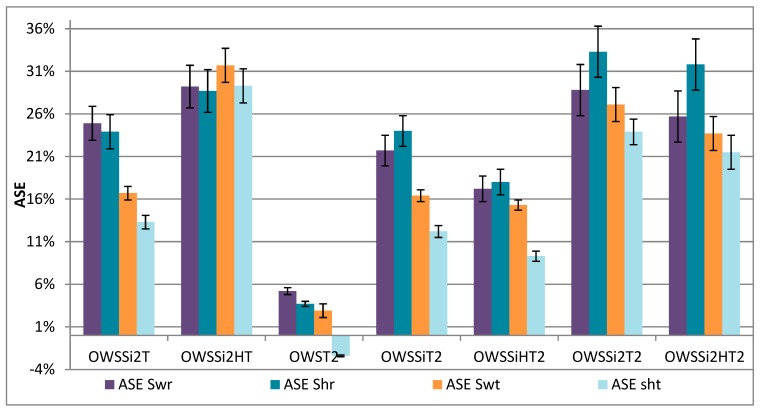
Anti swelling (ASE Sw r and ASE Swt) or shrinkage (ASE Sh r and ASE Sht) efficiency (dimensional stability) in the radial direction (r) and in the tangential direction (t) for the treated oven dried spruce wood using the modified treatments.

Figure 7 shows the swelling and shrinkage in both directions (radial and tangential) for oven-dried white spruce wood treated with modified treatments (increase in organosilanes concentration OWSSi2T and OWSSi2HT, change in heat treatment, OWSSiT2 and OWSSiHT2, and the two parameters combined, OWSSi2T2 and OWSSi2HT2).

**Figure 8:**
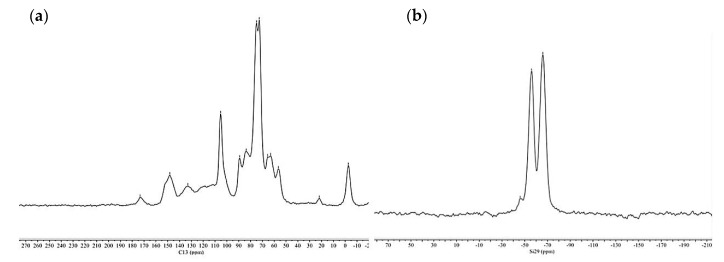
^13^C NMR and ^29^Si NMR spectra of organosilanes-treated oven dried wood (OWSSi2HT2) (**a**,**b**).

The new heat treatment alone (OWST2) has no impact on dimensional stability (<5%). The new heat treatment after impregnation of the organosilanes considerably reduces the dimensional stability (SD of 23% in the radial direction and 14% in the tangential direction) compared to the first heat treatment (Figure 6: 45% radial and 22.5% tangential). This is interesting because the swelling of the treated wood was more important. One hypothesis is that there would be a better incorporation of organosilanes into the cell wall, but with less hydroxyl condensation. This is due to a too low heat treatment at 50 °C, which does not favor the condensation step. The step at 103 °C is not sufficient afterwards for complete condensation and this creates a greater presence of hydroxyls than with the initial heat treatment. This is confirmed by the ^29^Si NMR spectrum (Figure 8), showing similar peaks observed for the T2 and T3 forms of organosilanes, but a new peak appears at -46 ppm, which corresponds to the T1 form (Figure 3). The presence of this peak shows that the OWSSi2HT2 samples had more hydroxyls present in the wood than the first organosilanes treatment on wood (OWSSiHT). The ^13^C NMR spectrum showed the same spectrum as that raw spruce wood but with a slight shift (0.4 ppm) for the peaks corresponding to carboxyl groups from lignin at 173.4 ppm, aryls groups from lignin at 133.0 ppm, and the C6 from cellulose crystalline at 65.4 ppm due to the bonding created between wood and organosilanes.

For the treatment with increase concentration of organosilanes (OWSSi2T and OWSSi2HT), the treatment is more effective with the wet step therefore similar conclusion than with the first treatment (OWSSiHT vs. OWSSiT). Compared to the initial treatment, swelling and shrinkage decreased in the radial direction (28% for OWSSi2HT versus 45% for OWSSiHT). However, the interesting fact is that it increased in the tangential direction (30.5% for Si2HT against 22.5% for OWSSiHT). The average dimensional stability (radial and tangential) is 30%, thus slightly lower than the original treatment (OWSSiHT at 35%).

The increase in organosilanes had no effect on overall dimensional stability, but rather on its distribution and condensation in the wood, changing the effect of swelling and shrinkage in both directions, improving in the tangential direction to the detriment of the radial direction. Finally, the last treatment, a combination of the two previous ones (concentration increase and modification of the heat treatment OWSSi2T2 and OWSSi2HT2) does not bring any improvement compared to the treatment with only an increase in the concentration of the organosilanes. The organosilanes treatment could be modified by adding some nanoparticles to improve the effect of dimensional stability.

## 4. Conclusions

White spruce wood with different initial moisture content was impregnated with organosilanes and then subjected to various post-treatments (day storage at 80%MC and thermal treatment). The presence of organosilanes was confirmed by infrared spectra and ^13^C and ^29^Si NMR showed organosilanes bonded to each other and to different wood polymers (cellulose, hemicellulose and lignin). By scanning electron microscopy with X-ray dispersive energy microanalysis, this study showed that organosilanes do not densify wood. Some lumens were filled with organosilanes, but in small quantities. In fact, they were more present in the cell wall and homogeneously.

In dimensional stability tests, green wood treated with organosilanes (41% of humidity) did not performed as well as the organosilanes-treated oven or air-dried wood. In all treatments, two were observed to be the best (OWSSiHT and AWSSiT). These two treatments showed the same performance, which means an improvement in dimensional stability in the radial direction of 45% (swelling and shrinkage) and in the tangential direction of 22.5% (swelling and shrinkage). In general, dimensional stability was improved by 35% in both directions. In addition, the maximum water vapor adsorption was reduced by 25% for organosilanes-treated wood relative to raw wood. 

For the modified treatments, only the treatment increasing the concentration of organosilanes (OWSSi2HT) showed an increase in dimensional stability in the tangential direction (30.5%) to the expense of the radial direction (29%). Dimensional stability in both directions has slowly decreased. Moreover, the cost of the process would increase due to the double quantity of MTMOS to be used. 

Finally, in this study, the treatment was effective on oven-dried and air-dried wood. The most interesting treatment was OWSSiHT for oven-dried wood and AWSSiT for air-dried wood, with a dimensional stability of white spruce wood of 35% and water vapor sorption decreasing by 25%. Nowadays, nanomaterials are used to improve some physical properties. So, to improve the dimensional stability of the organosilanes-treated wood, nanomaterials could be added to the treatment. For siding application, this treated wood must be able to be coated and nailed and must be UV resistant. New tests must therefore be performed to validate the use of this organosilanes-modified spruce wood.

## Figures and Tables

**Table 1:** Description of the 3 different factors used, creating 12 different white spruce treatments.

Factor 1	Factor 2	Factor3	Abbreviations
Organosilanes	Humidity Step	Heat Treatment	
Without organosilanes treatment	–	Thermal treatment, 95 °C for 24 h	T
	One day (24 h) storage at 80% RH and 20 °C	Thermal treatment, 95 °C for 24 h	HT
	–	Thermal treatment, 50 °C for 24 h then at 103 °C for 18 h	T2
organosilanes solution (MTMOS/ethanol/acetic acid): (0.12/1/0.005)	–	Thermal treatment, 95 °C for 24 h	SiT
	One day (24 h) storage at 80% RH and 20 °C	Thermal treatment, 95 °C for 24 h	SiHT
	–	Thermal treatment, 50 °C for 24 h then at 103 °C for 18 h	SiT2
	One day (24 h) storage at 80% RH and 20 °C	Thermal treatment, 50 °C for 24 h then at 103 °C for 18 h	SiHT2
organosilanes solution (MTMOS/ethanol/acetic acid): (0.24/1/0.005)	–	Thermal treatment, 95 °C for 24 h	Si2T
	One day (24 h) storage at 80% RH and 20 °C	Thermal treatment, 95 °C for 24 h	Si2HT
	–	Thermal treatment, 50 °C for 24 h then at 103 °C for 18 h	Si2T2
	One day (24 h) storage at 80% RH and 20 °C	Thermal treatment, 50 °C for 24 h then at 103 °C for 18 h	Si2HT2

**Table 2:** Maximal water vapor sorption (WVS) for the different treated woods and the percentage of improvement compared to raw wood.

	WVS Max	ΔWVS	%Improvement
Oven dried white spruce (14% MC)			
OWS	18.2%	0.2%	-
OWST	17.1%	0.3%	6%
OWSHT	18.3%	0.4%	0%
OWSSiT	14.2%	0.3%	22%
OWSSiHT	13.6%	0.2%	25%
Air dried white spruce (19% MC)			
AWS	17.8%	0.3%	-
AWST	18.4%	0.3%	-3%
AWSHT	18.2%	0.0%	-2%
AWSSiT	13.5%	0.1%	24%
AWSSiHT	14.0%	1.2%	22%
Green white spruce (41% MC)			
GWS	20.6%	0.1%	-
GWST	20.2%	0.8%	2%
GWSHT	19.5%	0.7%	5%
GWSSiT	16.7%	0.6%	19%
GWSSiHT	15.9%	0.5%	23%

## References

[1] Guy-Plourde S., Blanchet P., De Blois M., Robichaud F., Barbuta C. (2018). Cladding Materials in Non-Residential Construction: Consideration Crietria for Stakeholder in the Province of Quebec. J. Facade Des. Eng..

[2] Esteves B.M., Pereira H.M. (2009). Wood Modification by Heat Treatment: A review. Bioresources.

[3] Behkta P., Niemz P. (2003). Effect of High Temperature on the Change in Color, Dimensional Stability and Mechanical Properties of Spruce Wood. Holzforschung.

[4] Ahmed S.A., Morén T., Sehlstedt-Persson M., Blom A. (2017). Effect of oil impregnation on water repellency dimensional stability and mold susceptibility of thermally modified European aspenand downy birch wood. J. Wood Sci..

[5] Lekounougou S., Kocaefe D. (2014). Effect of thermal modification temperature on the mechanical properties, dimensional stability, and biological durability of black spruce (*Picea mariana*). Wood Mater. Eng..

[6] Rassam G., Taghiyari H.R., Ghofrani M., Jamnani B. (2011). Mechanical performance and dimensional stability of nano-silver impregnated densified spruce wood. Holz Als Roh Werkst..

[7] Welzbacher C.R., Wehsner J., Rapp A.O., Haller P. (2008). Thermomechanical densification combined with thermal modification of *Norway spruce* (*Picea abis* Karst) in industrial scale—Dimensional stbility an durability aspects. Holz Als Roh Werkst..

[8] Cai X., Blanchet P. (2011). Effect of vaccum time, formulation and nanoparticles on properties of surface-densified wood products. Wood Fiber Sci..

[9] Sandberg D., Kutnar A., Mantanis G. (2017). Wood Modification Technologies—A review. IForest Biogeosci. For..

[10] Brelid L.P., Simonson R., Risman P.O. (1999). Acetylation of solid wood using microwave heating Part 1: Studies of dielectric properties. Holz Als Roh Werkst..

[11] Pepin S., Blanchet P., Landry V. (2019). Performances of white pine and white spruce treated with organic fungicides using an aqueous buffered amine oxide preservation system. Bioresources.

[12] Bayani S., Taghiyari H.R., Papadopoulos A.N. (2019). Physical and Mechanical Properties of Thermally-Modified Beech Wood Impregnated with Silver Nano-Suspension and Their Relationship with the Crystallinity of Cellulose. Polymers.

[13] Papadopoulos A.N., Bikiaris D.N., Mitropoulos A.C., Kyzas G.Z. (2019). Nanomaterials and chemical modificaitions for enhanced keywood properties: A review. Nanomaterials.

[14] Fufa S.M., Hovde P.J. (2010). Nano-Based Modifications of Wood and Their Environmental Impact: Review. http://support.sbcindustry.com/Archive/2010/june/Paper_356.pdf.

[15] Xie Y., Hill C.A.S., Xiao Z., Militz H., Mai C. (2010). Silane Coupling agents used for natural fiber/polymer composites: A review. Compos. Part A.

[16] Cappelletto E., Maggini S., Girardi F., Bochicchio G., Tessadri B., Di Maggio R. (2013). Wood surface protection with different alkoxysilanes: A hydrophobic barrier. Cellulose.

[17] Wang X., Chai Y., Liu J. (2013). Formation of highly hydrophobic wood surfacces using silica nanoparticles modified with long-chain alkylsilane. Holzforschung.

[18] Donath S., Militz H., Mai C. (2004). Wood modification with alkoxysilanes. Wood Sci. Technol..

[19] De Vetter L., Van Den Bulcke J., Van Acker J. (2010). Impact of Organosilicon Treatments on the Wood-water Relationship of Solid Wood. Holzforschung.

[20] Giudice C.A., Alfieri P.V., Canosa G. (2013). Siloxanes synthesized “in situ” by sol-gel process for fire control in wood of Araucaria Angustifolia. Fire Saf. J..

[21] Hung K.-C., Wu J.-H. (2016). Characteristics and thermal Decomposition Kinetics of Wood-SiO2 Composites Derived by the sol-gel Process. Holzforschung.

[22] Broda M., Majika J., Olek W., Mazela B. (2018). Dimensional Stability and Hygroscopic Properties of Waterlogged Archaeological Wood Treated with Alkoxysilanes. Int. Biodeterior. Biodegrad..

[23] Hung K.-C., Wu J.-H. (2018). Comparison of Physical and Thermal Properties of Various Wood-inorganic Composites (WICs) Derived by the sol-gel Process. Holzforschung.

[24] Xie Y., Hill C.A.S., Sun D., Jalaludin Z., Wang Q., Mai C. (2011). Effects of dynamic aging (hydrolysis and condensation) behaviour of organofunctional silanes in the aqueous solution on their penetrability into the cell walls of wood. Bioresources.

[25] Lu Y., Feng M., Zhan H. (2014). Preparation of SiO_2_-wood composites by an ultrasonic-assisted sol-gel technique. Cellulose.

[26] Miranda I., Sousa V., Ferreira J., Pereira H. (2017). Chemical characterization and extractives composition of heartwood and sapwood from *Quercus faginea*. PLoS ONE.

[27] Robles E., Csoka L., Labidi J. (2018). Effect of reaction conditions on the surface modification of cellulose nanofibrils with aminopropyl triethoxysilane. Coatings.

[28] Siuda J., Perdoch W., Mazela B., Zborowska M. (2019). Catalyzed reaction of Cellulose and Lignin with Methyltrimethoxysilane—FT-IR, ^13^C NMR and ^29^Si NMR studies. Materials.

[29] Rao J., Zhou Y., Fan M. (2018). Revealing the interface structure and bonding mechanism of coupling agent treated WPC. Polymers.

[30] Sugahara Y., Okada S., Sato S., Kuroda K., Kato C. (1994). ^29^Si-NMR study of hydrolysis and initial polyconensation processes of organoalkoxysilanes. II Methyltriethoxysilane. J. Non Cryst. Solids.

[31] Baur S.I., Easteal A.J. (2013). Improved photoprotection of wood by chemical modification with silanes: NMR and ESR studies. Polym. Adv. Technol..

[32] Alma M.H., Hafizo[gtidle]lu H., Maldas D. (1996). Dimensional Stability of Several Wood Species Treated with Vinyl Monomers and Polyethylene Glycol-1000. Int. J. Polym. Biomater..

[33] Meier E. (2015). Wood! Identifying and Using Hundreds of Woods Worldwide, Wood Database. https://www.wood-database.com/.

[34] Brochier Salon M.-C., Belgacem M.N. (2011). Hydrolysis-condensation kinetics of different silane coupling agents. Phosphorus Sulfur Silicon.

[35] Bak M., Molnar F., Németh R. (2019). Improvement of dimensional stability of wood by silica nanoparticles. Wood Mater. Sci. Eng..

[36] Canosa G., Alfieri P.V., Giudice C.A. (2018). Low density wood impregnation water-repellent organosilicic compounds. J. Mater. Sci. Chem. Eng..

[37] Rowell R.M., Pettersen R., Han J.S., Rowell J.S., Tshabalala M.A. (2005). Cell Wall Chemistry. Handbook of Wood Chemistry and Wood Composite.

